# What could cause the reactivation of Epstein–Barr virus in individuals with long COVID

**DOI:** 10.1080/22221751.2025.2552712

**Published:** 2025-08-25

**Authors:** Jiaxin Ling, Jinlin Li

**Affiliations:** a Department of Medical Biochemistry and Microbiology, Uppsala University, Uppsala, Sweden; b Department of Medical Biochemistry and Microbiology, Zoonosis Science Center, Uppsala University, Uppsala, Sweden

**Keywords:** Long COVID, EBV, herpesvirus, SARS-CoV-2, COVID-19

**Dear Editor**

A significant number of COVID-19 patients have been subjected to wide-ranging persistent health problems after acute SARS-CoV-2 infection, known as long COVID. The pathophysiological mechanisms of long COVID are not fully understood; however SARS-CoV-2 persistent infection, activation of latent infection virus, immune dysregulation, and organ dysfunction are proposed as potential mechanisms [1]. Reactivation of Epstein–Barr virus (EBV), a tumour virus, has been noticed in the long COVID patients [2, 3], which could act as a key factor contributing to this syndrome. However, how EBV is reactivated in the context of SARS-CoV-2 infection is enigmatic. In this study, we investigated several possibilities that could result in EBV reactivation by the direct or indirect effects of SARS-CoV-2 infection, including (i) whether the infection of SARS-CoV-2 directly stimulates the activation of EBV in the epithelial cells; (ii) whether the substance, such as viral antigens and (pro) inflammatory factors, released from the SARS-CoV-2-infected epithelial cells lead to the reactivation of EBV in B cells; (iii) whether heme could result in the reactivation of EBV in B cells.

EBV switches its cellular tropisms between B cells and epithelial cells to effectively build infections. The latently infected B cells are thought to be the reservoir of the virus, while epithelial cells act as the site of productive infection during EBV infection in humans. EBV could also latently infect epithelial cells [4,5]. SARS-CoV-2 has a broad spectrum of cell tropism with an effective infection in epithelial cells but not B cells. Therefore, epithelial cells are the common cells that SARS-CoV-2 and EBV could coinfect. To examine if SARS-CoV-2 infection could cause EBV reactivation in epithelial cells, we used AGS-Bx1 (EBV latently infected epithelial cell lines) stably expressing ACE2 (AGS-Bx1-ACE2) as a cell model. AGS-Bx1-ACE2 cells were infected by the SARS-CoV-2 prototype strain. The expression of SARS-CoV-2 spike (S) protein was gradually increased from 24 h to 72 h post-infection (Figure 1(a)), suggesting SARS-CoV-2 can infect and replicate in AGS-Bx1-ACE2 cells. The expression of the transactivator of the EBV lytic cycle, BZLF1, did not exhibit a significant increase at different time points post SARS-CoV-2 infection at mRNA and protein levels (Figure 1(b,c)), demonstrating no reactivation of EBV. This conclusion was further supported by the results that there were no significant upregulation of EBV lytic early gene, BMRF1, DNA replication, and newly synthesized viral particles after SARS-CoV-2 infection (Figure 1(d–f)). Collectively, the data from our epithelial cell model indicate that direct SARS-CoV-2 infection does not stimulate EBV reactivation.

**Figure 1.**
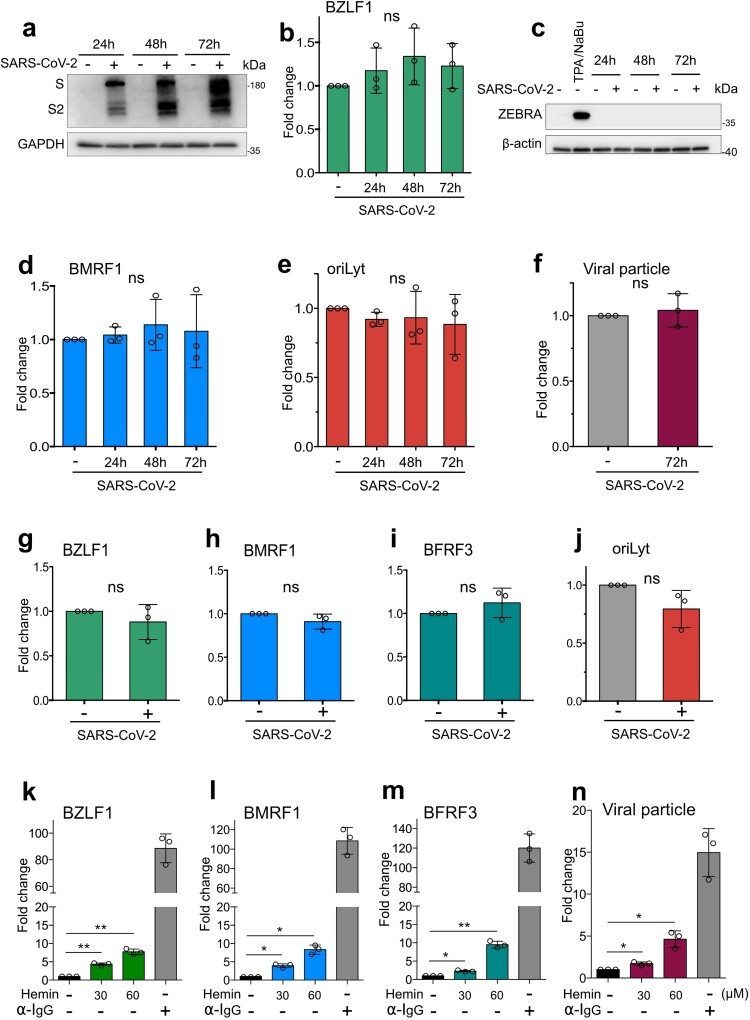
Heme results in the reactivation of EBV. AGS-Bx1-ACE2 cells were infected by the SARS-CoV-2 prototype strain (MOI = 1) (a–f). (a) The infection of SARS-CoV-2 on AGS-Bx1-ACE2 was evaluated by monitoring the expression of SARS-CoV-2 S protein using western blot. The transcript expression of EBV (b) lytic activator (BZLF1) and (d) early gene (BMRF1) were assessed by qPCR using specific primers. (c) The expression of ZEBRA (encoded by BZLF1) was examined by western blot. TPA/NaBu treatment was utilized as a positive control for the induction of EBV reactivation. (e) EBV DNA replication was investigated by qPCR using the specific primers targeting the oriLyt element. (f) The amount of EBV genome isolated from the DNAase-treated supernatant (72 h post-infection), which can reflect the number of viral particles, was quantified by qPCR using the specific primers targeting BMRF1. Vero E6 cells were infected by SARS-CoV-2 (MOI = 1) in the insert of the transwell. Around 24 h post-infection, Vero E6 cells started to show cytopathic effect (CPE). Akata-Bx1 cells were added to the bottom of the transwell and co-cultured with Vero E6 (g–j). After the co-culture for 48 h, Akata-Bx1 cell pellets were harvested and the lytic transcripts, BZLF1 (g), BMRF1 (h), BFRF3 (i) and DNA replication (j) were assessed by qPCR. Different concentrations (30 µM and 60 µM) of hemin were added to Akata-Bx1 cells (k–n). After 24 h, the expression of lytic transcripts BZLF1 (k), BMRF1 (l), and BFRF3 (m) were examined by qPCR. (n) The supernatants harvested at 48 h post treatments were used to quantify viral particles released. The data was shown as mean ± SD of three independent experiments. Statistical analyses were performed by one-way ANOVA (b, d, e, k–n) or Student’s t-test. *p ≤ 0.05; **p ≤ 0.01; ns, not significant.

SARS-CoV-2 appears unable to directly infect B cells due to the lack of ACE2 expression [6]. However, the viral antigens released from the infected cells could be uptaken by B cells through secreted exosomes [7]. Several SARS-CoV-2 proteins have been demonstrated as stimuli for the activation of NLRP3-mediated inflammatory reaction. Additionally, the pro-inflammatory factors, such as high-mobility group protein 1 released from SARS-CoV-2-infected Vero E6 cells [8], could trigger the activation of NLRP3 inflammasome, which might lead to the reactivation of EBV [9]. Therefore, to explore whether the substance released from SARS-CoV-2-infected epithelial cells leads to the reactivation of EBV in B cells, Vero E6 and Akata-Bx1 cells were co-cultured and were infected by SARS-CoV-2. Akata-Bx1 is the Burkitt lymphoma (BL) cell line latently infected with recombinant EBV and widely used as the EBV-infected B cells model. The markers of the EBV lytic phase were assessed by qPCR. There was no significant increase of EBV immediate early gene (BZLF1), early gene (BMRF1), DNA replication, and late gene (BFRF3) (Figure 1(g–j)). The results suggest that SARS-CoV-2-infected Vero E6 cells do not stimulate EBV reactivation from latently infected B cells. It is worth noting that there might be more complex interplays between SARS-CoV-2 infected cells and latently infected B cells *in vivo* because other cells, such as different kinds of immune cells, may directly or indirectly participate in [10], highlighting a comprehensive evaluation of the effects of SARS-CoV-2 infection on the EBV-infected B cells is needed in the future study.

Accumulating evidence suggests that persistent SARS-CoV-2 infection could act as a trigger for long COVID. Based on the results mentioned above, the reactivation of EBV in individuals with long COVID does not appear to result from the direct infection of SARS-CoV-2 in epithelial cells, nor from the released SARS-CoV-2 antigens or other inflammatory factors that are taken up by latently infected B cells. A recent study pointed out that heme was increased in persons with 6-month long COVID compared to the healthy and recovered ones [2]. EBV reactivation could be induced by heme modulation during the malaria infection [11]. To investigate whether heme could trigger the reactivation of EBV, we treated Akata-Bx1 cells with hemin (the oxidized form of heme) or with anti-human IgG as a positive control. As expected, anti-human IgG robustly activated the lytic cycle of EBV, evidenced by a dramatical increase of immediate early (BZLF1), early (BMRF1), late (BFRF3) lytic transcripts expression, and viral production (Figure 1(k–n)). Hemin treatment also led to a significant upregulation of lytic transcripts expression and an increase in the production of viral particles in a dose-dependent manner (Figure 1(k–n)), suggesting that EBV was reactivated by hemin. Besides, we treated the AGS-Bx1-ACE2 cells with hemin. Intriguingly, hemin treatment failed to induce a significant increase of EBV BZLF1, BMRF1, and BFRF3 at 24 h and 48 h post treatments in AGS-Bx1-ACE2 cell model (Figure S1(a–c)), indicating hemin can only trigger EBV reactivation in the B cell model.

In addition to the long COVID period, heme is elevated in the patients during acute SARS-CoV-2 infection [12]. The incidence of EBV reactivation is also increased in COVID-19 patients and is positively associated with the severity of COVID-19 [13]. What factors drive EBV reactivation during acute SARS-CoV-2 infection and in the long COVID stage? Do these stages share common underlying mechanisms, and could heme be a contributing factor in both? Further studies are needed to address these interesting questions. In addition, what are the different clinical consequences of EBV reactivation in the context of SARS-CoV-2 acute infection and the long COVID? Whether the upregulation of heme from acute SARS-CoV-2 infection triggers the reactivation of EBV, which enhances the expression of ACE2 [14], and thereby promotes SARS-CoV-2 infection, ultimately resulting in severe COVID-19, remains to be studied. Additionally, whether enhanced heme makes individuals with long COVID more susceptible to reinfection by SARS-CoV-2 is another concern. Some of the most common symptoms in persons with long COVID, such as fatigue, could also be caused by EBV infection. Whether the reactivation of EBV contributes to long COVID and the consequences of the increased incidence of EBV reactivation in individuals with long COVID in the long term require a comprehensive study, as EBV infection is associated with many chronic diseases.

## Author contributions

J. Ling and J.L. contributed equally to the conceptualization, investigation, writing of the original draft, and editing.

## Supplementary Material

Supplemental Material

## Data Availability

The data that support the findings of this study are available from the corresponding author upon reasonable request.
