# Supplementary material for: What could cause the reactivation of Epstein–Barr virus in individuals with long COVID
Source: Emerg Microbes Infect. 2025 Aug 25;14(1):2552712. doi: 10.1080/22221751.2025.2552712 (PMC12424145; doi:10.1080/22221751.2025.2552712)
Supplement: Supplemental Material [file TEMI_A_2552712_SM9978.docx]

**Supplementary Materials**

**What could cause the reactivation of Epstein-Barr virus in individuals with long COVID**

**Authors**

Jiaxin Ling ^1, 2^ and Jinlin Li ^1, 2^

**Affiliations**

1 Department of Medical Biochemistry and Microbiology, Uppsala University, Uppsala, Sweden.

2 Department of Medical Biochemistry and Microbiology, Zoonosis Science Center, Uppsala University, Uppsala, Sweden.

**Keyword: Long COVID, EBV, Herpesvirus, SARS-CoV-2, COVID-19**

**Correspondence: Jinlin Li,** [**jinlin.li@imbim.uu.se**](mailto:jinlin.li@imbim.uu.se)

**This file includes:** Materials and Methods part and Figure S1

**MATERIALS AND METHODS**

**Reagents, cell lines, and virus**

The following chemicals and antibodies were used in this study. Chemicals:

Sodium butyrate (NaBu, B5887, Sigma-Aldrich); 12-O-tetradecanoylhporbol-13-acetate (TPA, 4174, Sigma-Aldrich); Hemin (Cat#HY-19424, MedChemExpress); DNase I (M6101, Promega). Antibodies: mouse anti-EBV BZLF1 (sc-53904, 1:1000, Santa Cruz); GAPDH Monoclonal antibody (60004-1-Ig, Proteintech); Goat anti-Mouse IgG (H+L) Secondary Antibody, HRP (31430, Invitrogen); rabbit polyclonal anti-human IgG (A0423, DAKO, Glostrup Denmark). Akata-Bx1 and AGS-Bx1 cell lines were kindly provided by Prof. Maria Masucci from Karolinska Institutet. To construct the AGS-Bx1 cells stably expressing ACE2, lentivirus was packaged by co-transfection of FLVX-ACE2, which was constructed by cloning the CDS of human ACE2 to PLVX-IRES-Puro Vector (Clontech, TaKaRa) at Xhol and Xbal Restriction sites, with the 2nd generation lentiviral system plasmids psPAX2 and pMD2G (psPAX2 and pMD2G were a gift from Didier Trono Addgene plasmid #12260 and # 12259). Lentivirus-transduced AGS-Bx1 cells were selected under 1.5 µg/ml puromycin for at least 2 weeks and the expression of ACE2 was evaluated by western blot using an antibody against ACE2. Vero E6 cells were from ATCC (CRL-1586). The SARS-CoV-2 prototype strain used in this study was directly isolated form the COVID-19 patient [1].

**Quantitative real-time PCR**

Total RNA was extracted from cell pellets using RNeasy Mini Kit (74104, Qiagen). Around 1 µg RNA was used for the reverse transcription (RT) by High-Capacity Reverse transcription kit (4368813, Applied Biosystem) according to the attached protocol. The cDNA generated through RT was utilized for quantitative real-time PCR (qPCR) using Power Track SYBR Green Master Mix (A46109, Applied Biosystem) with cycling program: 95°C for 30 sec, followed by 40 cycles of denaturation at 95°C for 10 sec and annealing/extension at 60°C for 60 sec. Melt curve analysis was added by running from 65 °C to 95 °C with 0.5 °C increments at 5 sec/step. The specific primers for qPCR used in this study are: BZLF1 (F-5´GAAAATGCCGGGCCAAGTTT3´; R-5´TGTCCGGGGGATAATGGAGT 3´); BMRF1 (F-5´CAACACCGCACTGGAGAG; R-5´GCCTGCTTCACTTTCTTGG3´); BFRF3 (F-5´GCCATAGACAAGAGGCAGAG; R-5´CGG AGG CTGCTAATAGATGA3´); oriLyt gene (F-5’TCCTCTTTTTGGGGTCTC TG3’; R-5’CCCTCCTCCTCTCGTTATCC3’) and human GAPDH (F-5´TGGGCTACACTGAGCACCAG3´; R-5´ GGGTGTCGCTGTTGAAGTC3´). The primers used for detecting BMRF1, BFRF3, and OriLyt were described in our previous study [2]. Fold change was caculated as 2-Δ(ΔCt).

**Induction of the productive cycle and quantification of virus released in the supernatant**

Akata-Bx1 cells were induced to lytic cycle by incubation with rabbit polyclonal anti-human IgG (1:100) at 37°C for 1.5 h with shaking every 15 min and cells were continued to culture until harvest. AGS-Bx1 cells were induced by culture in medium supplemented with 30 ng/mL 12-O-tetradecanoylphobol-13-acetate (TPA) and 0.5 mM sodium butyrate (NaBu). The amount of virus released into supernatant from induced cells was quantified by qPCR. Briefly, the supernatant was subjected to the centrifugation at 14,000 rpm for 5 min and followed by treatment of 20 U/mL DNase I (M6101, Promega) to remove viral free DNA. EBV genome DNA in viral particles was extracted by DNeasy Blood & Tissue Kit (69504, Qiagen) and quantified by qPCR using Power SYBR Green PCR Master Mix (A46109, Applied Biosystem) using specific primers targeting BMRF1.

**Immunoblotting**

Immunoblotting was performed following standard procedures as described in our previous study [3].

**Figure S1.** AGS-Bx1-ACE2 cells were treated with hemin (60µM). Cells were harvested at 24h and 48h post treatments and the expression of BZLF1(**a**), BMRF1(**b**), and BFRF3(**c**) were assessed by qPCR. The data was shown as mean ± SD of three independent experiments. Statistical analyses were performed by one-way ANOVA. ns, not significant.

**References**

1. Ling, J., et al., *Infectious SARS-CoV-2 is rarely present in the nasopharynx samples collected from Swedish hospitalized critically ill COVID-19 patients.* Ir J Med Sci, 2023. **192**(1): p. 227-229.

2. Li, J., et al., *A single phosphoacceptor residue in BGLF3 is essential for transcription of Epstein-Barr virus late genes.* PLoS Pathog, 2019. **15**(8): p. e1007980.

3. Ling, J., et al., *A Heparan Sulfate Mimetic RAFT Copolymer Inhibits SARS-CoV-2 Infection and Ameliorates Viral-Induced Inflammation.* Adv Sci (Weinh), 2024: p. e2411737.
